# Association between dietary and circulating folate markers and kidney stones in American adults: A cross-sectional NHANES analysis

**DOI:** 10.1097/MD.0000000000048951

**Published:** 2026-05-29

**Authors:** Mohammad Safargar, Abbas Basiri, Hamed Kord-Varkaneh

**Affiliations:** a Student Research Committee, Tabriz University of Medical Sciences, Tabriz, Iran; b Urology and Nephrology Research Center, Research Institute for Urology and Nephrology, Shahid Labbafinejad Medical Center, Shahid Beheshti University of Medical Sciences, Tehran, Iran.

**Keywords:** cross-sectional studies, folic acid, kidney calculi, nutrition surveys, risk factors

## Abstract

The relationship between folate status and kidney stone disease (KSD) is poorly understood, despite folate’s essential role in one-carbon metabolism, which is related to pathways of stone formation. Given the limited and inconsistent findings from previous research, this study aimed to comprehensively investigate the association between both dietary folate intake and multiple circulating folate biomarkers with the prevalence of KSD in a large, nationally representative US adult population. We conducted a cross-sectional analysis of 9208 adult participants from the National Health and Nutrition Examination Survey, 2017 to 2020. Dietary folate was assessed using a 24-hour recall. Circulating biomarkers included total serum folate, 5-methyltetrahydrofolate, tetrahydrofolate (THF), and red blood cell (RBC) folate. Multivariable logistic regression models were used to estimate odds ratios and 95% confidence intervals for KSD across quartiles of each folate measure, adjusting for potential confounders. The overall weighted prevalence of KSD was 9.4%. In unadjusted models, higher RBC folate and THF concentrations were associated with increased odds of KSD. However, after adjusting for sociodemographic, lifestyle, and clinical confounders (Model 3), no significant associations were observed between dietary folate intake (Q4 vs Q1: odds ratio 0.94, 95% confidence interval: 0.57–1.55) or any circulating folate biomarkers (RBC folate, 5-methyltetrahydrofolate, serum folate, and THF) and the prevalence of KSD. Furthermore, no significant linear or nonlinear dose-response trends were detected. In this large, nationally representative study using rigorous survey-weighted analysis, folate status was not independently associated with the prevalence of kidney stone disease. These findings suggest that folate intake and metabolism may not be major drivers of stone formation in the general US adult population.

## 1. Introduction

Kidney stones are one of the most prevalent urological conditions, affecting approximately 11% of the population in the United States.^[1]^ The recurrence incidence is notably concerning, with as many as 50% of patients encountering recurrence within 5 years.^[2]^ The elevated prevalence and recurrence rate considerably impact patients’ health and quality of life while also exerting a financial strain on the healthcare system.^[3]^ The annual economic burden of kidney stones in the United States is estimated to be approximately 2 billion dollars.^[4]^ Moreover, alterations in dietary and lifestyle practices are expected to contribute to the ongoing increase in the incidence of kidney stones.^[5]^

The pathogenesis of KSD is multifactorial, with calcium oxalate (CaOx) stones comprising approximately 75% of all cases.^[6]^ Urinary oxalate concentration plays a pivotal role in CaOx supersaturation and crystallization, with endogenous synthesis accounting for the majority (approximately 60% to 80%) of the daily urinary oxalate load.^[7]^ This underscores the importance of metabolic pathways regulating oxalate production, particularly those involving folate-dependent one-carbon metabolism.^[8]^

In recent years, the role of B vitamins, including folate, in metabolic pathways relevant to kidney stone formation has garnered scientific interest.^[9–11]^ Folate, in its active coenzyme form, 5-methyltetrahydrofolate (5-MTHF), is integral to one-carbon metabolism, facilitating the transfer of one-carbon units essential for DNA synthesis and amino acid metabolism.^[12]^ Suboptimal folate status has been hypothesized to impair metabolic processes, potentially leading to increased oxalate production and a heightened risk of KSD development.

Additionally, folate status affects the metabolism of homocysteine. Deficiencies in folate or vitamin B12 can result in hyperhomocysteinemia, a condition associated with vascular and renal injury.^[13,14]^ Elevated homocysteine levels have been shown to exert direct toxic effects on renal tubular epithelial cells, mediated by oxidative stress, inflammation, and ferroptosis, thereby contributing to kidney damage.^[15]^

Despite a compelling biochemical rationale, epidemiological studies examining the relationship between folate status and KSD have been limited, yielding conflicting results. Research has predominantly focused on vitamin B6, with 1 study suggesting a protective association between high vitamin B6 intake and reduced stone risk in women, and 2 studies finding no association.^[16–18]^ Similarly, a study on vitamin B12 indicated a complex, potentially inverse relationship with KSD prevalence,^[10]^ while another study showed that genetically predicted circulating vitamin B12 levels were causally associated with a higher risk of KSD.^[11]^

To our knowledge, no large-scale population-based study has simultaneously evaluated the association between kidney stones and a comprehensive panel of specific circulating folate forms (including red blood cell [RBC] folate, 5-MTHF, and tetrahydrofolate [THF]) alongside dietary intake. National Health and Nutrition Examination Survey (NHANES) provides a robust platform for such analyses, offering detailed data on various folate forms, including total serum folate, RBC folate, 5-MTHF, and THF, measured using advanced methodologies such as isotope-dilution liquid chromatography, tandem mass spectrometry.^[19]^ Therefore, this study aimed to leverage the NHANES data to examine the associations between dietary folate intake and circulating folate biomarkers, total serum folate, 5-MTHF, THF, and RBC folate, and the prevalence of KSD in a nationally representative sample of US adults. By identifying these relationships, this study aims to contribute to a deeper understanding of the role of folate in kidney stone pathogenesis and inform potential dietary and clinical strategies for the prevention of KSD. This study hypothesized that lower dietary and circulating folate status would be associated with a higher prevalence of KSD.

## 2. Materials and methods

### 2.1. Study population

The NHANES is a cross-sectional, nationally representative survey designed to gather comprehensive data on the health and nutritional status of the noninstitutionalized US population. The study protocols were approved by the National Center for Health Statistics Research Ethics Review Board (ERB Protocols #2011-17 and #2018-01), and all participants provided written informed consent.

This analysis specifically utilized data from the 2017 to March 2020 Pre-Pandemic Dietary Data – Continuous NHANES public release. The study sample was derived from participants with complete information on the main variables of interest: history of kidney stones and dietary and circulating folate status. Additionally, individuals reporting implausible total energy intakes (<500 or >3500 kcal/day for women and <800 or >4000 kcal/day for men) were excluded from the analysis to ensure data quality.^[20]^

**Figure 1.**
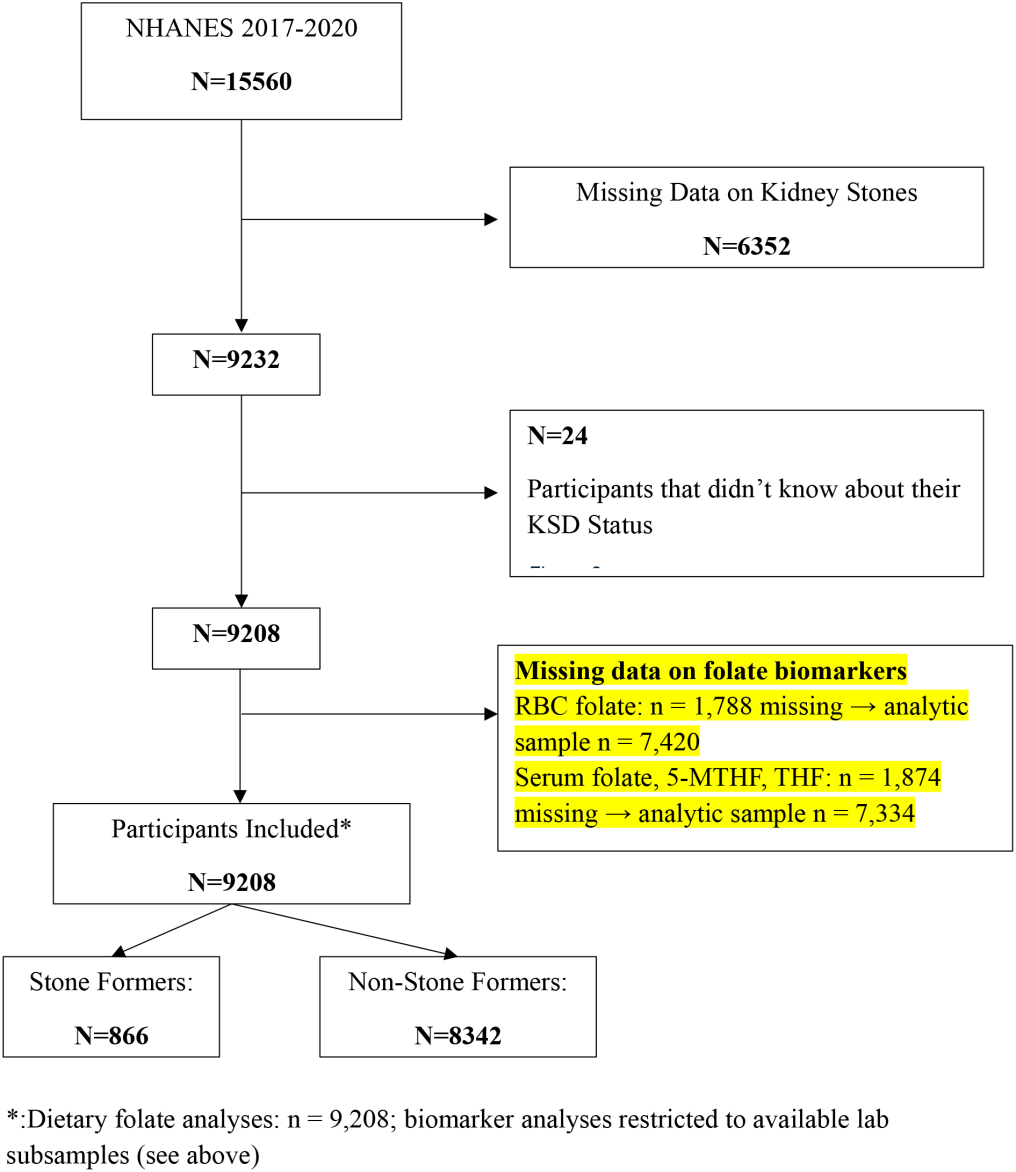
Flowchart of participant selection from the NHANES 2017 to March 2020 Pre-pandemic cycle. 5-MTHF = 5-methyltetrahydrofolate, KSD = kidney stone disease, NHANES = National Health and Nutrition Examination Survey, RBC = red blood cell, THF = tetrahydrofolate.

Consequently, the final analytical sample for the dietary analysis comprised 9208 participants. Because of standard missingness in NHANES laboratory blood draws, circulating biomarker models were conducted on available subsamples: 7420 participants for RBC folate (1788 missing) and 7334 participants for total serum folate, 5-MTHF, and THF (1874 missing), as detailed in the participant flow diagram (Fig. 1).

### 2.2. Dietary folate intake

Dietary data in the NHANES were collected through two 24-hour dietary recall interviews. The first interview was administered in person at the Mobile Examination Center (MEC), and the second was conducted via telephone 3 to 10 days later. To enhance data quality and minimize the potential for recall bias, only data from the first dietary recall interview were used to estimate folate intake. Our analysis focused solely on folate derived from food sources (including naturally occurring food folate and synthetic folic acid from fortified foods), with supplementary intake excluded from the analysis. Dietary folate intake is expressed as total folate in micrograms per day (μg/d). For the statistical analysis, participants were categorized into quartiles based on their total dietary folate intake, with the lowest quartile (Q1) designated as the reference category.

### 2.3. Circulating folate variables’ status

Circulating folate status was assessed using biomarker concentrations measured in blood samples collected during MEC examinations. The specific biomarkers used were total serum folate, 5-MTHF, THF, and RBC folate. For analysis, each circulating biomarker was independently divided into quartiles, with the first quartile (Q1) serving as the reference group for association testing.

### 2.4. Outcome variable

The main outcome variable in this study was the history of kidney stone disease (KSD). KSD status was determined via self-report using participants’ affirmative response to the standardized survey question: “Have you ever had kidney stones?” Individuals who answered “yes” were categorized as having a history of KSD.

### 2.5. Covariates

The following variables were included as covariates in the multivariable regression models to account for potential confounding: age, sex, race/ethnicity, educational level, family poverty-to-income ratio, smoking history, drinking status, physical activity, body mass index (BMI), total energy intake, dietary calcium intake, dietary caffeine intake, hypertension, and diabetes mellitus. Race/ethnicity was categorized into 4 groups: Mexican American, non-Hispanic White, non-Hispanic Black, and Other race/ethnicity (including multiracial).

Smoking status was determined by participant self-report and categorized based on the frequency of current smoking into 3 discrete groups: “Every Day,” “Some Days,” and “Not At All,” with the latter serving as the reference group. Alcohol consumption was assessed as a dichotomous variable (yes/no), indicating whether the participant reported consuming alcoholic drinks in the past 12 months, based on a question regarding average alcoholic drinks consumed per day.

### 2.6. Statistical analysis

All statistical analyses were performed using SPSS software (version 27; IBM Corporation), incorporating appropriate sample weights, strata, and primary sampling units to account for the complex multistage probability sampling design of NHANES. Examination sample weights (WTMECPRP) were applied to produce nationally representative estimates. Continuous variables are presented as weighted means ± standard errors, and categorical variables are presented as weighted percentages. Differences in baseline characteristics were assessed using the complex samples general linear model for continuous variables and the Rao–Scott χ^2^ test for categorical variables.

Multivariable **survey-weighted logistic regression models** were employed to estimate odds ratios (ORs) and 95% confidence intervals (CIs). Folate intake and related biomarkers were categorized into quartiles, with the lowest quartile (Q1) serving as the reference category. Three distinct models were developed to calculate the ORs and corresponding 95% CIs. Model 1 was the crude, unadjusted model. Model 2 was adjusted for age, sex, and BMI. Model 3 included adjustments for a comprehensive set of potential confounders: age, sex, BMI, total energy intake, race/ethnicity, educational level, family poverty-to-income ratio, smoking history, drinking status, physical activity, dietary calcium intake, dietary caffeine intake, hypertension, and diabetes mellitus. Statistical significance was set at *P* < .05 for all tests.

Because folate biomarkers were assessed in the full MEC sample during the 2017 to March 2020 cycle rather than a designated subsample, the standard MEC examination weights (WTMECPRP) were appropriately applied for all dietary and biomarker analyses to ensure unbiased, nationally representative estimates.

## 3. Results

### 3.1. Baseline characteristics of participants

This study included 9208 adults from the NHANES, among whom 866 (9.4%) reported a history of kidney stones. The demographic, anthropometric, and clinical characteristics of the study population stratified by kidney stone history are detailed in Table 1.

**Table 1:** Characteristics of NHANES participants based on the presence of kidney stone.

Variable	Stone formers (n = 866)	Non-stone formers (n = 8342)	*P*-value
	Mean ± SE or N (%)	Mean ± SE or N (%)	
Weight (kg)†	88.08 ± 1.20	83.81 ± 0.56	<.001†
Age	53.74 ± 0.77	47.81 ± 0.51	<.001
BMI (kg/m^2^)	31.23 ± 0.42	29.64 ± 0.16	<.001
Sex (% M)	52.8%	47.6%	.175
Race/ethnicity* (%):			
Mexican American	6.4	8.5	<.001*
Other Hispanic	8.0	7.6	<.001
Non-Hispanic White	70	61.8	<.001
Non-Hispanic Black	6.7	12	<.001
Other races – including multiracial	8.8	10.1	<.001
Tap water source (%):			
Community supply	64.7	65	.224
Don’t drink tap water	21.3	20.1	.224
Other	12.6	12	.224
Don’t know	1.4	2.9	.224
Waist circumference (cm)	104.87 ± 0.92	100.14 ± 0.46	<.001
5-methyltetrahydrofolate	38.40 ± 1.77	37.81 ± 0.84	.686
Tetrahydrofolate (nmol/L)	0.81 ± 0.038	0.75 ± 0.019	.089
Serum total folate (ng/mL)	18.26 ± 0.88	17.89 ± 0.43	.607
RBC folate (ng/mL)	577.46 ± 15.54	531.70 ± 7.86	.001
Total plain water drank yesterday (g)	1301.57 ± 60.03	1238.84 ± 27.31	.302
Total tap water drank yesterday (g)	613.87 ± 48.04	641.98 ± 28.87	.636
Total bottled water drank yesterday (g)	687.69 ± 55.28	596.85 ± 30.20	.105
Diabetes, %			
Yes	19.5	10.7	<.001
No	77.2	86.8	<.001
Prediabetes	3.3	2.4	<.001
Don’t know	0	0.1	<.001
Hypertension, %			
Yes	49.7	30.8	<.001
No	50.3	69.1	<.001
Don’t know	0	0.1	<.001
Smoking status, %			
Every day	24.8	31.1	.152
Some days	10	8.7	.152
Not at all	65.2	60.2	.152
Alcohol status (average alcoholic drinks/d – past 12 mo), %			
1–3 drinks	85.3	80.3	.278
4–9 drinks	13.3	17.2	.278
10 drinks or more	1.4	2.4	.278
Refused	0	0	.278
Don’t know	0	0.1	.278

BMI = body mass index, NHANES = National Health and Nutrition Examination Survey, SE = standard error.

* Chi-square test for categorical variables.

† General linear model used for continuous variables.

Participants with a history of kidney stones were significantly older (mean [standard error]: 53.74 ± 0.77 vs 47.81 ± 0.51 years, *P* < .001) and exhibited higher mean anthropometric measurements, including weight (88.08 ± 1.20 vs 83.81 ± 0.56 kg, *P* < .001), BMI (31.23 ± 0.42 vs 29.64 ± 0.16 kg/m^2^, *P* < .001), and waist circumference (104.87 ± 0.92 vs 100.14 ± 0.46 cm, *P* < .001). The stone-former group had a higher proportion of males (52.8% vs 47.6%, *P* = .175) and non-Hispanic White individuals. Furthermore, the prevalence of comorbidities such as diabetes (19.5% vs 10.7%, *P* < .001) and hypertension (49.7% vs 30.8%, *P* < .001) was significantly higher in the group with a history of kidney stones.

In the unadjusted comparison of folate biomarkers, participants with kidney stones had significantly higher circulating concentrations of RBC folate (577.46 vs 531.70 ng/mL, *P* = .001). No significant baseline differences were observed in 5-MTHF, THF, or total serum folate levels.

### 3.2. Dietary nutrient intake

The comparison of dietary intakes between the groups is shown in Table 2. Overall, the dietary profiles were largely similar. No statistically significant differences were observed in the intake of energy, macronutrients, dietary fiber, total folate, or key minerals such as calcium and magnesium. However, individuals with a history of kidney stones reported a significantly lower vitamin B6 intake (1.93 vs 2.15 mg, *P* = .023).

**Table 2:** Nutrients and energy intake among patients with and without kidney stones.

Variable	Stone formers (n = 866)	Non-stone formers (n = 8342)	*P*-value*
	Mean ± SE	Mean ± SE	
Energy (kcal/d)	2185.08 ± 63.41	2173.01 ± 14.79	.859
Protein (g/d)	80.55 ± 2.51	81.68 ± 0.83	.678
Carbohydrate (g/d)	245.06 ± 7.04	245.66 ± 1.93	.275
Total fats (g/d)	89.75 ± 2.88	89.63 ± 0.74	.969
Dietary fiber, total (g/d)	15.99 ± 0.71	16.56 ± 0.30	.351
Total sugars (g/d)	112.14 ± 4.55	105.58 ± 1.46	.180
Total saturated fatty acids (g)	29.87 ± 1.00	29.20 ± 0.29	.561
Total polyunsaturated fatty acids (g)	20.73 ± 0.86	20.89 ± 0.26	.840
Cholesterol (mg)	325.25 ± 15.95	318.44 ± 4.75	.678
Vitamin E as alpha-tocopherol (mg)	9.25 ± 0.31	9.41 ± 0.12	.607
Total folate (μg)	373.9 ± 13.73	371.67 ± 4.57	.875
Vitamin B6 (mg)	1.93 ± 0.06	2.15 ± 0.04	**.023**
Vitamin C (mg)	69.82 ± 4.91	76.01 ± 1.65	.206
Vitamin D (D2 + D3; μg)	4.29 ± 0.31	4.32 ± 0.14	.939
Iron (mg)	14.50 ± 0.48	13.84 ± 0.15	.225
Zinc (mg)	10.51 ± 0.28	10.94 ± 0.12	.175
Magnesium (mg)	295.58 ± 8.55	303.23 ± 3.09	.371
Calcium (mg)	990.83 ± 27.49	947.36 ± 12.88	.213
Caffeine (mg)	190.37 ± 29.33	168.10 ± 5.11	.464
Alcohol (g)	9.02 ± 1.44	11.58 ± 0.53	.086

Bold value indicates statistical significance (*P* < .05).

SE = standard error.

* General linear model was used.

### 3.3. Multivariate association between folate status and kidney stone risk

The survey-weighted ORs for kidney stones across the dietary folate and folate biomarker quartiles are presented in Table 3. In the unadjusted analysis (Model 1), higher quartiles of RBC folate and THF were significantly associated with increased odds of kidney stones. However, these associations were attenuated in Model 2 (adjusted for age, sex, and BMI). In the fully adjusted model (Model 3), no significant associations were observed between any quartile of dietary folate, RBC folate, 5-MTHF, total serum folate, or THF and the prevalence of kidney stones. Furthermore, no significant linear dose-response trends were detected for any folate marker (all *P*-trend > .05).

**Table 3:** Odds ratios and confidence intervals (95%) for folate status variables across folate status quartiles.

	Variable quartiles				*P*-trend
	Q1	Q2	Q3	Q4	
Odds ratios (95% CI) for kidney stone					
Folate intake (μg/d)					
Cutoff (No. of participants)	≤ 62 (N = 3732)	63–123 (N = 3086)	124–213 (N = 2806)	≥214 (N = 2768)	
Model 1	1	0.823 (0.598–1.134)	0.764 (0.598–1.059)	1.011 (0.725–1.410)	.968
Model 2	1	0.802 (0.596–1.079)	0.727 (0.536–0.988)	0.998 (0.735–1.356)	.955
Model 3	1	0.778 (0.477–1.268)	0.725 (0.446–1.177)	0.943 (0.574–1.546)	.865
RBC folate (ng/mL)					
Cutoff (No. of participants)	≤ 381 (N = 2061)	382–481 (N = 1984)	482–608 (N = 1774)	≥609 (N = 1601)	
Model 1	1	1.138 (0.713–1.816)	1.620 (1.058–2.482)	1.496 (1.126–1.988)	<.001
Model 2	1	1.073 (0.675–1.705)	1.449 (0.934–2.250)	1.230 (0.932–1.622)	.021
Model 3	1	0.964 (0.511–1.818)	1.362 (0.722–2.572)	0.880 (0.552–1.401)	.909
5-methyltetrahydrofolate (nmol/L)					
Cutoff (No. of participants)	≤ 23.4 (N = 1866)	23.5–34.5 (N = 1818)	34.6–51.0 (N = 1865)	≥51.1 (N = 1785)	
Model 1	1	0.616 (0.363–1.045)	0.883 (0.569–1.372)	1.035 (0.690–1.552)	.635
Model 2	1	0.643 (0.385–1.073)	0.882 (0.566–1.375)	1.044 (0.697–1.564)	.696
Model 3	1	0.617 (0.366–1.039)	0.848 (0.460–1.565)	0.882 (0.508–1.533)	.812
Total serum folate (ng/mL)					
Cutoff (No. of participants)	≤ 11 (N = 1874)	12–16 (N = 1803)	17–23.5 (N = 1850)	≥23.6 (N = 1807)	
Model 1	1	0.716 (0.413–1.242)	0.950 (0.627–1.438)	1.005 (0.677–1.492)	.759
Model 2	1	0.743 (0.439–1.258)	0.945 (0.621–1.437)	0.994 (0.685–1.442)	.861
Model 3	1	0.660 (0.385–1.133)	0.879 (0.479–1.614)	0.782 (0.458–1.338)	.534
Tetrahydrofolate (nmol/L)					
Cutoff (No. of participants)	≤ 0.47 (N = 1683)	0.48–0.67 (N = 1803)	0.68–0.91 (N = 1889)	≥0.92 (N = 1959)	
Model 1	1	1.054 (0.606–1.833)	1.021 (0.726–1.437)	1.620 (1.164–2.255)	**.002**
Model 2	1	1.007 (0.581–1.746)	0.916 (0.659–1.273)	1.333 (0.946–1.878)	.079
Model 3	1	0.876 (0.458–1.673)	0.997 (0.648–1.534)	1.084 (0.677–1.734)	.626

Bold value indicates statistical significance (*P* < .05).

Model 1: crude model.

Model 2: adjusted for age, sex, and BMI.

Model 3: adjusted for age, sex, BMI, race/ethnicity, educational level, family poverty-to-income ratio (PIR), smoking history, drinking status, physical activity, dietary calcium intake, dietary caffeine intake, total energy intake, hypertension, and diabetes.

BMI = body mass index, CI = confidence interval.

## 4. Discussion

This large cross-sectional study using a nationally representative sample of US adults investigated the relationship between comprehensive folate markers and the prevalence of KSD. The principal finding of this study was that neither dietary folate intake nor circulating serum biomarkers (RBC folate, 5-MTHF, total serum folate) were independently associated with the prevalence of kidney stones after adjusting for sociodemographic and clinical confounders. The lack of a significant association in our fully adjusted models suggests that folate status may not be an independent determinant of kidney stone prevalence in the general population.

Our results align strongly with the recent Mendelian randomization study by Yang et al.^[11]^ Using genetic variants as instrumental variables to assess causality, Yang et al found that genetically predicted circulating folate levels were not associated with the risk of KSD (OR: 1.10, *P* = .507), despite identifying significant causal roles for vitamin B12 and zinc.^[11]^ This alignment between our cross-sectional observational data and their genetic causal inference provides convergent evidence that folate status within the physiological range is likely not a major driver of stone formation in the general population.

This pattern of “null results” for B-vitamins in general populations mirrors the historical trajectory of vitamin B6 research. While early prospective data from the *Nurses’ Health Study I* by Curhan et al suggested that high vitamin B6 intake was protective against stones in women, these findings were not replicated in men.^[17]^ More importantly, a subsequent, more comprehensive analysis by Ferraro et al, which pooled data from the *Health Professionals Follow-up Study* and *Nurses’ Health Studies I and II*, found no significant association between vitamin B6 intake and incident stones after multivariate adjustment.^[16]^ Our findings for folate appear to follow this same paradigm: while unadjusted models may hint at associations, rigorous control for confounding abolishes the effect.

In contrast to our null findings for folate, other recent NHANES analyses have identified associations for different B-vitamins, suggesting distinct lithogenic roles for specific micronutrients. Wu recently reported an L-shaped inverse association between dietary niacin (vitamin B3) and kidney stones, where higher intake was protective, particularly in adults under age 60.^[9]^ Similarly, Liu et al found that higher dietary vitamin B12 intake was associated with a reduced risk of stones, although their age-stratified analysis revealed complex, U-shaped relationships in younger adults.^[10]^ The discrepancy between these significant findings for B3 and B12 and our null finding for folate may be explained by the specific enzymatic paths each vitamin regulates. While niacin directly mitigates oxidative stress and inflammation via the NF-κB pathway,^[21]^ folate’s primary role in re-methylating homocysteine may be less critical for stone formation in a population where folate deficiency is rare due to mandatory food fortification.

The lack of an association in our study is biologically plausible when examining the specificity of oxalate metabolism. The primary metabolic pathway for endogenous oxalate synthesis involves the conversion of glyoxylate to oxalate by lactate dehydrogenase.^[22]^ The alternative detoxification pathway, converting glyoxylate to glycine, is dependent on the enzyme alanine-glyoxylate aminotransferase, which requires vitamin B6 (pyridoxal-5′-phosphate) as a cofactor, not folate.^[23–25]^ While folate deficiency can elevate homocysteine levels (a risk factor for oxidative stress),^[26,27]^ there is no direct evidence in human or animal models that folate deficiency independently shunts glyoxylate toward oxalate synthesis. Since even vitamin B6 – the direct cofactor for glyoxylate detoxification, has shown inconsistent associations with stone risk in large epidemiological cohorts, it is biologically consistent that folate, which operates upstream in a separate methylation cycle, would have a negligible impact on stone formation in a general population.

Furthermore, the “Threshold Effect” of mandatory fortification likely blunts any potential variation. The US population generally maintains adequate folate status due to fortified cereals and grain products. It is plausible that once a minimum physiological threshold is met to prevent overt metabolic dysfunction, additional folate intake confers no further benefit against crystallization. As demonstrated by Harb et al, it is micronutrient inadequacy (specifically regarding vitamin B6 and vitamin A) that appears to drive risk in recurrent stone formers, rather than variation within the normal range of folate.^[28]^

Initial unadjusted analyses in our study suggested a potential link between higher RBC folate and kidney stones. However, this association disappeared after adjusting for potential confounders, particularly age and BMI. This suggests that the crude association was likely driven by demographic factors, older adults and those with higher BMI are at higher risk for stones and may also possess distinct dietary or metabolic folate profiles. For example, older adults in the US often have higher serum folate levels due to widespread fortification and supplement use, while simultaneously having a higher cumulative risk of kidney stones due to age-related metabolic changes. Once these confounding factors were strictly controlled for in our Model 3, the independent “effect” of folate vanished.

The strengths of this study include the use of the NHANES dataset, which provides a large, nationally representative sample that enhances the generalizability of our findings. We also had access to detailed information on dietary intake, serum biomarkers, and a wide array of potential confounders, which allowed for robust statistical adjustments. Several limitations of this study must be acknowledged. First, the cross-sectional design prevents the establishment of causal or temporal relationships. We cannot distinguish whether folate status preceded or followed stone formation, nor can we rule out reverse causation, where patients may have altered their dietary habits following a KSD diagnosis. Second, kidney stone history was self-reported and lacked detail regarding stone composition (e.g., CaOx vs uric acid), recurrence, or time since diagnosis. Third, dietary intake was assessed using a single 24-hour recall, which may not accurately reflect long-term habitual intake. However, to mitigate this, we analyzed RBC folate, a robust indicator of long-term folate status (reflecting the past ~120 days). Moreover, despite rigorous adjustment for confounders, the possibility of residual confounding from unmeasured variables (such as specific urinary biochemical parameters or detailed supplement usage) cannot be ruled out. Finally, our dietary analysis focused exclusively on folate intake from food sources and did not account for dietary supplement use. Because circulating folate biomarkers reflect total systemic exposure derived from both diet and supplements, this discrepancy may partly explain the lack of perfect concordance between dietary intake and circulating status. Future studies should incorporate total supplemental folate intake to provide a more comprehensive assessment.

## 5. Conclusion

In conclusion, this large, nationally representative study found no significant association between dietary or circulating folate biomarkers and the prevalence of KSD after rigorous adjustment for confounders. These findings do not support a substantial role for folate in the etiology of kidney stones in the general US adult population. Future research, particularly longitudinal cohort studies and Mendelian randomization analyses, is warranted to confirm these null findings and definitively assess causality. Until such evidence is available, these results suggest that folate status is unlikely to be a primary nutritional concern for kidney stone prevention, and findings should be interpreted cautiously regarding clinical management.

## Acknowledgments

The authors thank the participants and staff of the NHANES for their valuable contributions.

## Author contributions

**Conceptualization:** Hamed Kord-Varkaneh, Abbas Basiri.

**Data curation:** Mohammad Safargar, Abbas Basiri.

**Formal analysis:** Mohammad Safargar, Hamed Kord-Varkaneh.

**Methodology:** Mohammad Safargar, Hamed Kord-Varkaneh.

**Supervision:** Hamed Kord-Varkaneh.

**Writing – original draft:** Mohammad Safargar, Abbas Basiri, Hamed Kord-Varkaneh.

**Writing – review & editing:** Abbas Basiri, Hamed Kord-Varkaneh.
